# *Haemophilus parainfluenzae* septic arthritis following orthopedic procedures in immunocompetent patients with diverse management: A case report of 2 rare cases

**DOI:** 10.1097/MD.0000000000046433

**Published:** 2026-05-12

**Authors:** Min-Shiue Ho, Chang-Hao Lin, Shih-Hao Chen, Yu-Sian Ding, Chen-Hao Chiang

**Affiliations:** a Department of Orthopaedics, Ditmanson Medical Foundation Chia-Yi Christian Hospital, Chia-Yi, Taiwan; b Department of Orthopaedics, Taichung Tzu Chi Hospital, Buddhist Tzu Chi Medical Foundation, Taichung, Taiwan; c Department of Microbiology, Immunology and Biopharmaceuticals, College of Life Sciences, National Chiayi University, Chia-Yi, Taiwan.

**Keywords:** arthroscopy, case report, *Haemophilus parainfluenzae*, periprosthetic joint infection, septic arthritis, shoulder arthroplasty

## Abstract

**Rationale:**

*H. parainfluenzae* is an uncommon cause of musculoskeletal infection, particularly after orthopedic procedures.

**Patient concerns:**

Two cases of *H. parainfluenzae* infection in immunocompetent patients following reverse shoulder arthroplasty and arthroscopic knee procedures.

**Diagnoses:**

*H. parainfluenzae* was identified as the causative pathogen through synovial fluid culture. Case 1 was identified as periprosthetic joint infection of the shoulder, and Case 2 as septic arthritis of the knee.

**Interventions:**

Case 1 was managed with needle aspiration and oral antibiotics. Case 2 underwent arthroscopic debridement and received antibiotic therapy.

**Outcomes:**

Both patients recovered without recurrence of infection following appropriate treatment. In Case 2, the patient’s C-reactive protein levels decreased from 18.3 mg/dL at presentation to 0.217 mg/dL by postoperative day 36, indicating resolution of inflammation and restoration of function.

**Lessons:**

These cases highlight the potential of *H. parainfluenzae* infection in healthy individuals and underscore the importance of accurate diagnosis and appropriate management to ensure favorable outcomes.

## 1. Introduction

Postoperative infection is a serious complication of orthopedic surgery, with rare microorganisms presenting unique diagnostic and therapeutic challenges. *Haemophilus parainfluenzae*, a facultatively anaerobic Gram-negative coccobacillus, is commonly observed as a commensal organism in the upper respiratory tract.^[1]^ It rarely causes deep tissue infections. However, under certain conditions, such as immunocompromise, surgical manipulation, or the presence of orthopedic implants, it can become an opportunistic pathogen capable of causing serious musculoskeletal infections, including septic arthritis and osteomyelitis.^[2–8]^

Diagnoses of infections caused by *H. parainfluenzae* may be delayed or even missed because of the rarity of this organism in musculoskeletal infections and because of its strict growth requirements (e.g., the need for factor V). Routine culture protocols may fail to isolate the organism, leading to false-negative results or delayed identification, which complicates clinical management.^[1]^ *H. parainfluenzae* produces β-lactamase enzymes that confer resistance to ampicillin and other common β-lactam antibiotics that are typically used empirically against orthopedic infections. Therefore, culture and susceptibility testing are essential to guide antimicrobial therapy.^[1]^ Studies on carbapenemase-encoding genes in other Gram-negative pathogens, such as Burkholderia cepacia and Aeromonas sobria, highlight the importance of understanding pathogen-specific resistance profiles to guide postoperative infection management.^[9]^

*H. parainfluenzae* is capable of biofilm formation, particularly because it naturally inhabits the supragingival plaque in the human oral cavity, with specific genes essential for aerobic and anaerobic biofilm growth identified through genomic studies.^[10]^ This genetic basis of biofilm formation highlights its adaptive strategies in the oral niche. Importantly, this biofilm-forming capability presumably contributes to its persistence and complicates its eradication in infections involving orthopedic implants,^[11]^ indicating the need for accurate pathogen identification and tailored treatment strategies. Studies on staphylococcal biofilms further highlight that biofilm-associated cells can exhibit markedly increased antimicrobial tolerance compared with planktonic cells, supporting the clinical relevance of biofilm-mediated persistence.^[12]^

Infections caused by *H. parainfluenzae* are uncommon and typically reported in association with endocarditis, respiratory tract infections, or invasive disease in immunocompromised hosts. Joint infections following orthopedic procedures are particularly rare, and the limited number of reported cases results in incomplete information regarding clinical presentation, antimicrobial susceptibility, management, and the role of biofilm-mediated persistence in implant-associated infections.^[1]^

In this report, we present 2 rare cases of *H. parainfluenzae* infection following orthopedic procedures: one after reverse shoulder arthroplasty and the other after arthroscopic knee release. The objective of this report is to highlight the diagnostic challenges, antimicrobial susceptibility considerations, and management strategies in rare orthopedic infections caused by *H. parainfluenzae*. These cases also illustrate the microbiological characteristics and therapeutic considerations associated with this uncommon but clinically important pathogen, including evidence of biofilm involvement supported by clinical findings and existing literature on its biofilm-forming capacity.

## 2. Case report

Only de-identified patient-specific information was used to ensure confidentiality. This study was conducted in accordance with the ethical principles outlined in the Declaration of Helsinki. The study design was approved by Ditmanson Medical Foundation Chia-Yi Christian Hospital institutional review board (approval no.: 2025048). The requirement for informed consent was waived by Ditmanson Medical Foundation Chia-Yi Christian Hospital institutional review board because of the retrospective nature of this study.

### 2.1. Case 1

An 87-year-old man underwent a reverse shoulder arthroplasty on the right side to address osteoarthritis and an irreparable rotator cuff tear with pseudoparalysis. He had no known immunosuppressive conditions or diabetes. He reported an allergy to intravenous ampicillin and ampicillin/sulbactam. His postoperative course was uneventful until 12 months later, when a protruding mass developed on his right shoulder. The patient denied experiencing any fever or shoulder pain. Given the absence of pain or systemic symptoms, initial considerations included sterile seroma, soft tissue mass, or chronic bursitis. Arthrocentesis revealed turbid yellowish pus, which microbiological culture revealed to be *H. parainfluenzae* using the VITEK-MS system (MALDI-TOF MS; bioMérieux, France), confirming periprosthetic joint infection.

**Figure 1.**
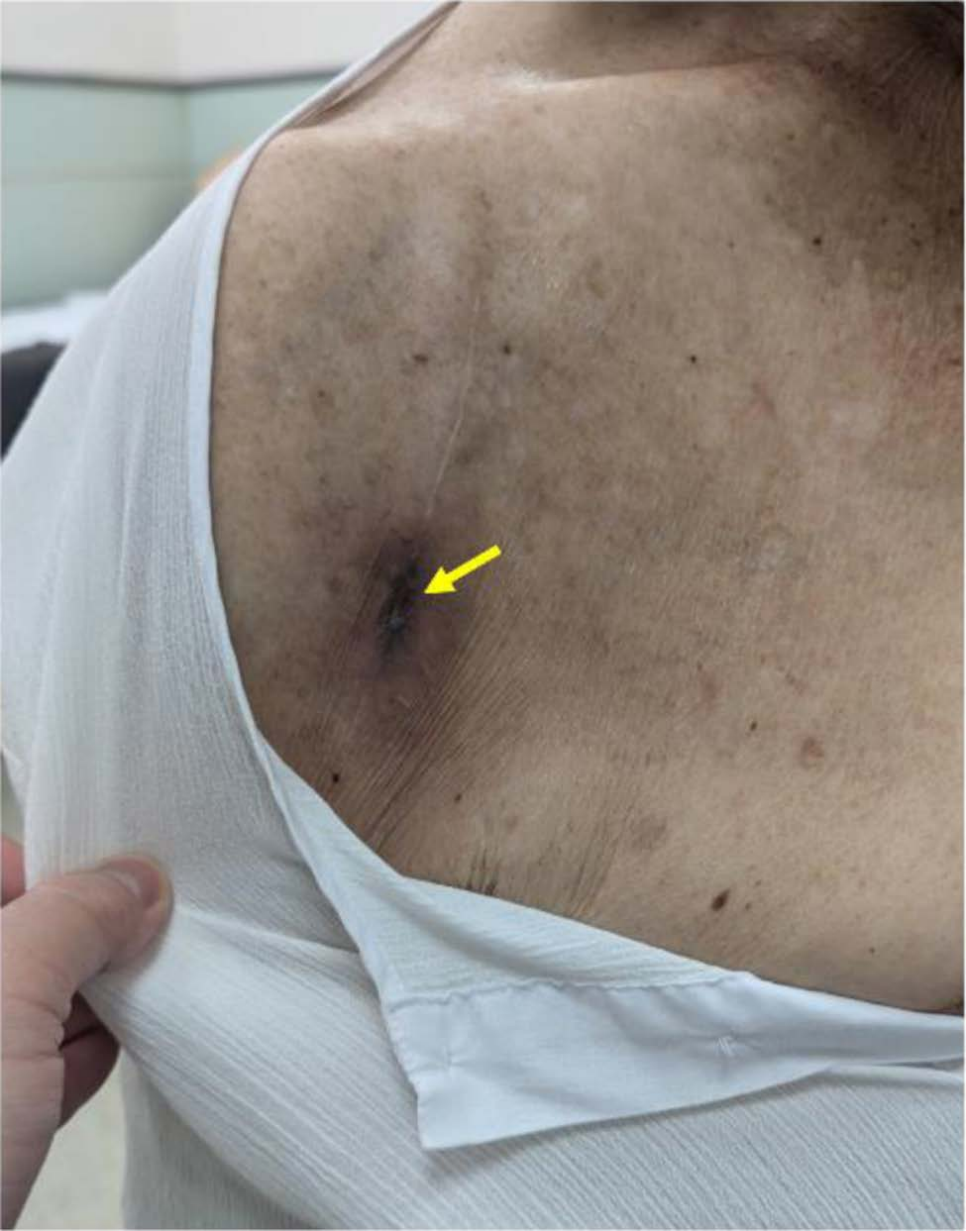
Protruding mass with turbid yellowish pus in an 87-yr-old man after reverse shoulder arthroplasty. The mass resolved after series needle aspiration and oral antibiotics. Yellow arrow indicates the protruding mass after improvement.

The patient was treated with series needle aspiration and a course of oral antibiotics. Based on the antimicrobial susceptibility profile determined by the Kirby–Bauer disk diffusion method, trimethoprim/sulfamethoxazole was initially chosen and administered for 2 weeks. This treatment was discontinued because the patient developed gastrointestinal discomfort, and minocycline was subsequently used orally for 6 weeks. The patient’s symptoms gradually disappeared with no pus formation observed after 2 months of therapy (Fig. 1). No adverse or unanticipated events were observed.

### 2.2. Case 2

A 42-year-old man without immunosuppressive conditions, diabetes, or known drug allergies presented with progressive stiffness and mild pain in the left knee during ambulation. He had undergone anterior cruciate ligament reconstruction on the same knee more than 10 years earlier at another hospital. Because of his persistently limited range of motion (0°–80°), he underwent arthroscopic fibrolysis, lateral retinaculum release, and surgical manipulation, which improved his postoperative range of motion to 0° to 145°.

Twenty-one days after surgery, the patient visited the emergency department with complaints of fever, left knee stiffness, swelling, pain, and wound discharge observed at home. His body temperature was 37.1°C, and he denied any symptoms of upper respiratory or urinary tract infection. Physical examination revealed swelling, tenderness, and redness of the left knee, but no warmth or discharge. It also revealed that the patient’s knee again had a limited range of motion. Given the postoperative timing, differential diagnoses such as sterile joint effusion, mechanical irritation, or infection with common organisms were initially considered. Laboratory tests revealed leukocytosis, with a white blood cell count of 11,760/μL and an elevated C-reactive protein (CRP) level of 18.3 mg/dL. Bedside ultrasonography revealed mild fluid accumulation in the left knee joint. Intra-articular aspiration revealed turbid yellowish fluid. Microbiological culture of the joint aspirate identified *H. parainfluenzae* using the VITEK-MS system, confirming septic arthritis.

**Figure 2.**
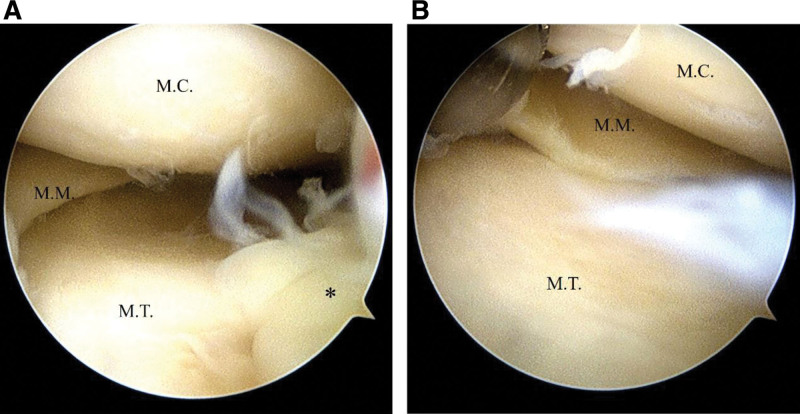
Septic arthritis with biofilm accumulation in a 42-year-old man’s left knee after arthroscopic fibrolysis. (A) Biofilm accumulation in the medial compartment. (B) After debridement. MC = medial condyle, MT = medial tibial plateau, MM = medial meniscus; asterisk, biofilm.

Two days after the patient visited the emergency department, arthroscopic examination, debridement, and biofilm debulking were performed (Fig. 2). For 2 weeks, the patient received parenteral ampicillin. Following the initial 2 weeks, the regimen was switched to the oral combination of ampicillin/sulbactam and rifampicin for another 6 weeks. The selection of both parenteral ampicillin and oral ampicillin/sulbactam was guided by in vitro susceptibility results confirmed via Kirby–Bauer disk diffusion testing. Rifampicin was included as part of combination therapy based on the treating physician’s clinical judgment for the postoperative joint infection. Postoperative inflammatory markers were closely monitored, with CRP levels measured at 2.702 mg/dL on postoperative day 6, 4.047 mg/dL on postoperative day 13, and 0.217 mg/dL on postoperative day 36. At that point, no clinical signs of infection were observed, and subsequent follow-up confirmed sustained resolution and preserved knee function. No adverse or unanticipated events were observed.

## 3. Discussion

Infections involving *H. parainfluenzae* are uncommon, and reports after orthopedic procedures are extremely rare. Our report contributes to the limited literature by describing 2 cases of postoperative joint infection in immunocompetent patients without traditional risk factors. These cases highlight that *H. parainfluenzae* can also cause clinically significant infections in otherwise healthy individuals and expand the spectrum of orthopedic scenarios in which this pathogen should be considered. Table 1 presents previously reported cases of *H. parainfluenzae* infection following orthopedic procedures. Most of these cases involved immunocompromised hosts or patients with identifiable risk factors, such as diabetes mellitus, chronic lymphocytic leukemia, asplenia, recent upper respiratory tract infection, prior wound complications, or dental abscesses. By contrast, our 2 cases involved immunocompetent patients without systemic comorbidities or earlier infections, indicating that clinicians should maintain a high index of suspicion for this pathogen even in low-risk populations.

**Table 1:** Summary of previously reported cases of *H. parainfluenzae* infection following orthopedic procedures.

Study	Age/sex	Site	Orthopedic procedure/infection onset time	Comorbidities	Blood tests	Treatment	Time to symptom resolution
Cheong and Moo^[13]^	92/F	Knee	Total knee arthroplasty/5 y	None	WBC: 12,700/μL, CRP: 3.36 mg/dL	Debridement, antibiotic therapy, and implant retention, IV ceftriaxone + ciprofloxacin for 7 d → oral ciprofloxacin + cotrimoxazole for 6 mo	Not reported
Medel-Plaza et al^[7]^	67/M	Knee	Total knee arthroplasty/not reported	Type 2 diabetes mellitus and hypertension	WBC: 12,000/μL, CRP: 1.3 mg/dL	Debridement, antibiotic therapy, and implant retention, IV ceftriaxone for 24 h + ciprofloxacin for 7 d → oral ciprofloxacin + cotrimoxazole for 6 mo	1 mo after debridement, antibiotic therapy, and implant retention
Baron et al^[4]^	17/M	Knee	Meniscus repair/25 d	Upper respiratory tract infection	WBC: 20,300/μL, CRP: 1.36 mg/dL, ESR: 34 mm/h	Arthroscopic irrigation and debridement (twice), Cefazolin perioperatively → IV ampicillin (2000 mg) every 6 h → amoxicillin for 21 d	6 weeks after initial meniscus repair
O’Neil et al^[8]^	56/F	Hip	Inter-articular steroid injection/6 d	Asplenia	WBC: normal, CRP: 2.3 mg/dL, ESR: 25 mm/h	Arthrotomy with synovectomy and irrigation, Ceftriaxone for 9 wk	Not achieved
Bailey et al^[3]^	75/M	Knee	Total knee arthroplasty/not reported	Chronic lymphocytic leukemia, prior wound complication	CRP: 4.6 mg/dL, ESR: 82 mm/h	Debridement + flucloxacillin and rifampicin for 6 wk (recurrence) → 2-stage revision with gentamicin and vancomycin cement + three-dose cefuroxime prophylaxis	Not reported
Jellicoe et al^[5]^	78/F	Hip	Total hip arthroplasty/11 yr	Dental extraction	WBC: 7400/μL, ESR: 88 mm/h	Flucloxacillin + ampicillin for 4 wk → 2-stage revision with gentamicin beads and cement spacer	6 weeks after revision surgery
Manian^[6]^	72/M	Knee	Knee arthroplasty/6 yr	Dental abscess	Not reported	Oral cephalexin for 6 mo → IV ceftriaxone for 8 wk → oral ciprofloxacin for 2 yr	Not reported
Auten et al^[2]^	74/M	Cervical spine	Cervical foraminectomy/20 yr	Tooth abscess removal	WBC: 7800/μL	Laminectomy, IV trimethoprim and sulfamethoxazole for 5 weeks + tobramycin for 2 weeks → oral ciprofloxacin for 1 week	4 months after laminectomy
Case 1	87/M	Shoulder	Reverse shoulder arthroplasty/12 mo	None	Not assessed	Series needle aspiration Trimethoprim and sulfamethoxazole for 2 weeks → minocycline for 6 weeks	2 months after antibiotic therapy
Case 2	42/M	Knee	Arthroscopic fibrolysis and lateral release/21 d	None	WBC: 11,760/μL, CRP: 18.3 mg/dL, ESR: 34 mm/h	Arthroscopic irrigation and debridement, Ampicillin for 2 weeks → Unasyn + rifampicin for 6 wk	36 days after antibiotic therapy

As shown in Table 1, the joints affected by *H. parainfluenzae* infection in previous cases varied, with the knee being the most common site, typically after procedures such as total knee arthroplasty or arthroscopic surgery. Other reported sites included the hip after intra-articular steroid injection and total hip arthroplasty and the cervical spine 2 decades after foraminotomy. Our cases involved the knee and shoulder joints, aligning with the general pattern but also expanding the diversity of the involved sites.

The pathogenesis of joint infections caused by *H. parainfluenzae* remains unclear. Hematogenous spread is regarded as a potential mechanism, particularly because our 2 cases and a previously reported case lacked typical risk factors such as trauma, oral infections, or a history of dental procedures.^[13]^ Given that *H. parainfluenzae* is part of the normal flora of the oral cavity,^[14]^ reactivation of a subclinical infection may also represent a potential mechanism, particularly in immunocompromised patients, in whom latent microorganisms may become pathogenic. Recent studies have provided mechanistic insights into the biofilm-forming capacity of H. parainfluenzae and its potential role in opportunistic infections. Metagenomic analyses of peri-implant oral biofilms identified H. parainfluenzae as a commensal capable of participating in stable multi-species biofilm communities, suggesting its ability to persist on implant surfaces.^[15]^ Functional genomics studies using transposon insertion sequencing further delineated essential and conditionally essential genes for growth under aerobic and anaerobic biofilm conditions, offering a genetic basis for its persistence and opportunistic pathogenicity outside the oral cavity.^[10]^ Complementary in vivo experiments using a chinchilla otitis media model confirmed that H. parainfluenzae forms biofilm communities containing both bacterial and host components, which promote bacterial persistence and resistance to clearance, emphasizing the potential clinical relevance of biofilm-mediated infections in implant- or joint-associated contexts.^[16]^

Previous reports have indicated that the interval between orthopedic intervention and symptom onset substantially varies across cases, from as early as 6 days up to several years (Table 1). In our context, Case 2 developed symptoms of infection 21 days after surgery, indicating an early onset. Previous reports have also commonly indicated elevated levels of inflammatory markers such as white blood cell count, CRP, and erythrocyte sedimentation rate. In our context, Case 2 had a markedly elevated level of CRP at 18.3 mg/dL, which gradually normalized in parallel with clinical improvement.

Diagnosing infections caused by *H. parainfluenzae* can be challenging. *H. parainfluenzae* requires enriched media such as chocolate agar and incubation in CO_2_ for optimal growth, and it may be overlooked in routine culture procedures.^[3]^ A review indicated that the majority of cases of *H. parainfluenzae* infection were identified through synovial fluid culture. Synovial fluid culture is the primary diagnostic method for confirming *H. parainfluenzae*. Blood cultures are also considered to be essential, given the presumed hematogenous spread of the pathogen.^[17]^ In the present cases, *H. parainfluenzae* was identified using the VITEK-MS system, which rapidly analyzes bacterial protein spectra and markedly shortens the turnaround time from isolation to species identification.

In the cases reported in Table 1, the treatment strategies used considerably varied, ranging from prolonged intravenous and oral antibiotic therapy to surgical debridement and implant revision. In these cases, the majority of patients underwent multiple interventions. In this present report, Case 2 was successfully managed with arthroscopic debridement, biofilm removal, and a sequential antibiotic regimen. This treatment led to symptom resolution within 36 days, indicating that early surgical intervention combined with appropriate antibiotic therapy may be effective in treating patients with such infections. Given the limited number of reported cases, the optimal antimicrobial regimen for joint infections caused by *H. parainfluenzae* remains unknown. Nevertheless, several classes of antibiotics have been used with favorable outcomes. For example, certain β-lactam antibiotics, such as ceftriaxone, cefazolin, ampicillin, levofloxacin, rifampicin combined with flucloxacillin, and trimethoprim combined with sulfamethoxazole, have demonstrated clinical effectiveness against *H. parainfluenzae* infection.^[8]^ In our 2 cases, the choice of antimicrobial agents was guided by clinical response and tolerability.^[18]^ Antimicrobial susceptibility indicated that the *H. parainfluenzae* strains were sensitive to ampicillin, ampicillin/sulbactam, cefuroxime, cefotaxime, meropenem, trimethoprim/sulfamethoxazole, and minocycline. The first patient, who experienced side effects from trimethoprim/sulfamethoxazole, was successfully treated with minocycline monotherapy. The second patient received an initial course of intravenous ampicillin, which was subsequently switched to oral ampicillin/sulbactam combined with rifampicin. Both patients demonstrated complete resolution of symptoms without any signs of recurrence, indicating that tailored antibiotic therapy based on individual factors may result in favorable outcomes in cases of joint infections caused by *H. parainfluenzae*. Treatment strategies in these cases thus ranged from non-surgical oral antibiotic therapy to surgical debridement with sequential antibiotics, with all regimens individualized according to susceptibility testing and clinical response. Notably, both infections were successfully managed without removal of the prosthesis or the fixation devices, underscoring that with timely diagnosis, surgical debridement, and susceptibility-guided antimicrobial therapy, implant retention may be a feasible strategy in selected patients.

This report has several limitations. First, as a case report of only 2 patients, the findings may not be generalizable. Second, blood cultures were not performed, which limits the ability to definitively rule out bacteremia or distant infectious sources. Finally, treatment was individualized based on clinical response and tolerance, so the outcomes may not be directly applicable to all patients.

Overall, this case report contributes to the literature on *H. parainfluenzae* infections following orthopedic procedures by demonstrating that *H. parainfluenzae* infections can occur in the absence of traditional risk factors and may respond well to prompt surgical and medical management. The 2 cases demonstrate the spectrum of management strategies, from non-surgical series joint aspiration with oral therapy to surgical debridement with sequential antibiotics, providing further guidance for diagnosing and treating this rare pathogen in orthopedic practice. These cases highlight key clinical points: *H. parainfluenzae* infection can occur even in healthy patients without typical risk factors; prompt synovial fluid culture and antimicrobial susceptibility testing are essential; antibiotic therapy should be guided by susceptibility results and patient tolerance; and early surgical intervention, when indicated, may improve outcomes. Given the rarity of *H. parainfluenzae* joint infections, further studies are needed to better understand the pathogenesis, optimal antimicrobial regimens, and long-term outcomes, which could help inform future guidelines for diagnosis and management.

## Acknowledgments

The authors thank the help from the Clinical Data Center, Ditmanson Medical Foundation Chia-Yi Christian Hospital for providing administrative and technical support. This study is based in part on data from the Ditmanson Research Database provided by Ditmanson Medical Foundation Chia-Yi Christian Hospital. The interpretation and conclusions contained herein do not represent the position of Ditmanson Medical Foundation Chia-Yi Christian Hospital.

## Author contributions

**Conceptualization**: Chen-Hao Chiang.

**Investigation**: Chang-Hao Lin, Yu-Sian Ding.

**Writing – original draft**: Min-Shiue Ho.

**Writing – review & editing**: Chang-Hao Lin, Shih-Hao Chen, Chen-Hao Chiang.
